# High levels of 5-hydroxymethylcytosine (5hmC) is an adverse predictor of biochemical recurrence after prostatectomy in *ERG*-negative prostate cancer

**DOI:** 10.1186/s13148-015-0146-5

**Published:** 2015-10-15

**Authors:** Siri H. Strand, Soren Hoyer, Anne-Sofie Lynnerup, Christa Haldrup, Tine Maj Storebjerg, Michael Borre, Torben F. Orntoft, Karina D. Sorensen

**Affiliations:** Department of Molecular Medicine, Aarhus University Hospital, Aarhus, Denmark; Department of Histopathology, Aarhus University Hospital, Aarhus, Denmark; Department of Urology, Aarhus University Hospital, Aarhus, Denmark

**Keywords:** Prostate cancer, DNA methylation, Epigenetics, 5-hydroxymethylation, 5hmC, ERG, Prognostic, Biomarker

## Abstract

**Background:**

Prostate cancer (PC) can be stratified into distinct molecular subtypes based on *TMPRSS2-ERG* gene fusion status, but its potential prognostic value remains controversial. Likewise, routine clinicopathological features cannot clearly distinguish aggressive from indolent tumors at the time of diagnosis; thus, new prognostic biomarkers are urgently needed. The DNA methylation variant 5-hydroxymethylcytosine (5hmC, an oxidized derivative of 5-methylcytosine) has recently emerged as a new diagnostic and/or prognostic biomarker candidate for several human malignancies. However, this remains to be systematically investigated for PC. In this study, we determined 5hmC levels in 311 PC (stratified by *ERG* status) and 228 adjacent non-malignant (NM) prostate tissue specimens by immunohistochemical analysis of a tissue microarray, representing a large radical prostatectomy (RP) cohort with long clinical follow-up. We investigated possible correlations between 5hmC and routine clinicopathological variables and assessed the prognostic potential of 5hmC by Kaplan-Meier and uni- and multivariate Cox regression analyses in *ERG+* (*n* = 178) vs*. ERG−* (*n* = 133) PCs using biochemical recurrence (BCR) as endpoint.

**Results:**

We observed a borderline significant (*p* = 0.06) reduction in 5hmC levels in PC compared to NM tissue samples, which was explained by a highly significant (*p* < 0.001) loss of 5hmC in *ERG−* PCs. *ERG* status was not predictive of BCR in this cohort (*p* = 0.73), and no significant association was found between BCR and 5hmC levels in *ERG+* PCs (*p* = 0.98). In contrast, high 5hmC immunoreactivity was a significant adverse predictor of BCR after RP in *ERG*− PCs, independent of Gleason score, pathological tumor stage, surgical margin status, and pre-operative prostate-specific antigen (PSA) level (hazard ratio (HR) (95 % confidence interval (CI)): 1.62 (1.15–2.28), *p* = 0.006).

**Conclusions:**

This is the first study to demonstrate a prognostic potential for 5hmC in PC. Our findings highlight the importance of *ERG* stratification in PC biomarker studies and suggest that epigenetic mechanisms involving 5hmC are important for the development and/or progression of *ERG−* PC.

**Electronic supplementary material:**

The online version of this article (doi:10.1186/s13148-015-0146-5) contains supplementary material, which is available to authorized users.

## Background

In 2012, more than 1 million men worldwide were diagnosed with prostate cancer (PC), accounting for 15 % of all male cancer diagnoses that year [1]. While most PCs are indolent and rarely progress into clinically significant disease, a subset of PC patients develop highly aggressive metastatic disease with lethal outcome [2]. At the time of diagnosis, currently available routine prognostic tools (mainly Gleason score (GS), serum prostate-specific antigen (PSA), and clinical tumor stage) are unable to accurately predict the outcome of PC. Accordingly, although widespread use of PSA testing has facilitated early detection of PC, it has also led to over diagnosis and overtreatment of many indolent tumors [3]. Thus, novel and improved prognostic biomarkers for PC are urgently needed.

Approximately 50 % of all PCs carry the *TMPRSS2-ERG* gene fusion [4] that places the proto-oncogene *ERG* under androgen regulation, resulting in overexpression of this ETS family transcription factor. However, there is conflicting evidence as to whether the *TMPRSS2-ERG* fusion and/or the level of *ERG* expression have prognostic implications [5]. Nevertheless, results from several studies indicate that distinct molecular mechanisms are at play in *ERG*-positive (*ERG+*) vs*. ERG*-negative (*ERG−*) PCs, including significant differences in the epigenome [6–14].

Methylation of the cytosine 5′ carbon (5-methylcytosine, 5mC) in CpG dinucleotides is the most extensively investigated epigenetic modification of the human genome. Whereas PC is characterized by a low frequency of somatic mutations, DNA methylation changes are considered one of its hallmarks [15], and several studies have shown that specific DNA methylation changes have promising potential as diagnostic and/or prognostic markers for PC [16–19]. However, the mechanisms of 5mC removal remained obscure until the recent discovery of the ten-eleven translocated (TET) proteins that convert 5mC to 5-hydroxymethylcytosine (5hmC) in an active oxidative demethylation process [20, 21]. 5hmC can be further oxidized by TETs to 5-formylcytosine (5fC) and 5-carboxylcytosine (5caC), which are removed by base excision repair and replaced by an unmodified cytosine to complete the demethylation process [22, 23]. Active DNA demethylation is recognized as an important mechanism of plasticity in epigenetic regulation. However, accumulating evidence indicates that 5hmC is not only simply an intermediate in the process of active demethylation but also may function as a distinct epigenetic mark [24]. Indeed, it has been demonstrated that 5mC and 5hmC interact with unique sets of chromatin binding proteins [25], suggesting that these epigenetic marks hold distinct roles in chromatin regulation and/or organization.

Because standard methods for DNA methylation analysis, such as genomic bisulfite sequencing, cannot distinguish between 5mC and 5hmC, the contribution of 5hmC to epigenetic regulation has been overlooked [26]. Recent reports indicate that 5hmC levels are relatively high in terminally differentiated cells and lower in stem/progenitor cells [27–29]. Consistent with this, several studies have reported significantly reduced levels of 5hmC in cancer compared to non-malignant (NM) tissue samples [27, 29–39], and reduced levels of 5hmC have been suggested as a potential diagnostic marker for malignant transformation in several cancer types, including PC [27, 31, 32, 34, 37, 39]. However, for the latter, this is currently based only on two small-scale studies, reporting reduced 5hmC immunoreactivity in 30 PC vs*.* 10 NM [27] and 11 PC vs*.* 11 NM prostate tissue samples [37], respectively. To the best of our knowledge, there are no previous reports of a prognostic potential of 5hmC in PC, but low 5hmC levels have been associated with poor outcome in melanoma [34], myelodysplastic syndromes [35], gastric cancer [38], and glioblastoma [29], while high 5hmC levels have been associated with poor outcome in acute myeloid leukemia (AML) [40].

In the present study, we investigated the level of 5hmC in 311 malignant and 228 NM prostate tissue samples from a large radical prostatectomy (RP) patient cohort with 80 months median follow-up. We used a commercially available polyclonal anti-5hmC antibody with validated performance and specificity for 5hmC, as demonstrated in several published studies [21, 27–29, 37, 38, 41–44]. Our results indicate that 5hmC levels are significantly reduced in *ERG−* but not in *ERG+* PCs, as compared to NM prostate tissue samples. Furthermore, we found that 5hmC had significant prognostic potential in *ERG−* but not in *ERG+* PCs.

## Results

### 5hmC levels are significantly reduced in *ERG−* PC tissue samples

**Fig. 1:**
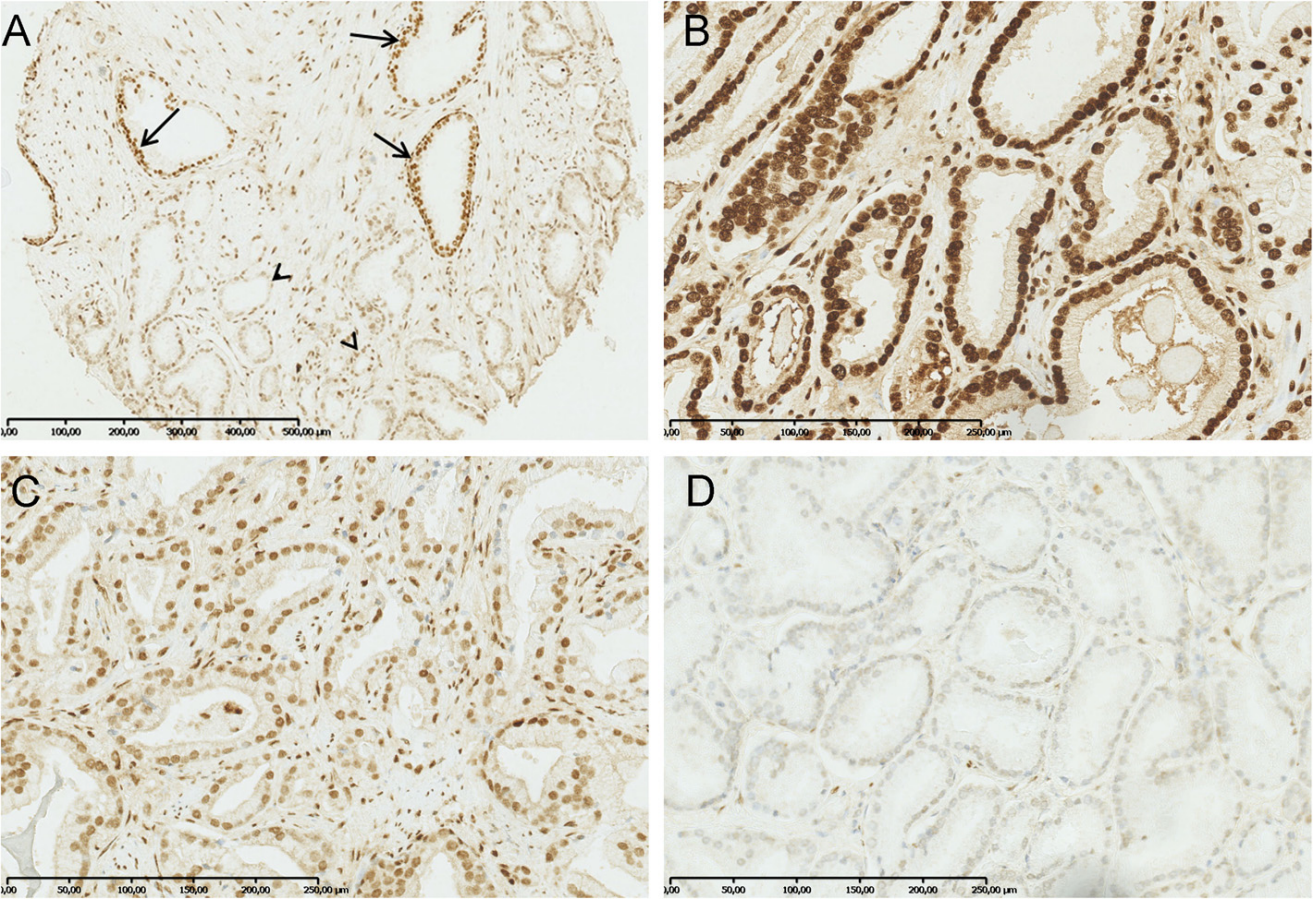
Representative images of 5hmC immunoreactivity in malignant and NM prostate tissue samples. **a** TMA tissue core (ERG− PC) containing both malignant and NM prostate glands. Reduced 5hmC levels were observed in the malignant (grade 1, *arrowheads*) compared to the NM glands (grade 2, *arrows*). **b** Strong 5hmC staining in malignant core (grade 2). **c** Intermediate 5hmC staining in malignant core (grade 1). **d** No 5hmC staining in malignant core (grade 0)

**Fig. 2:**
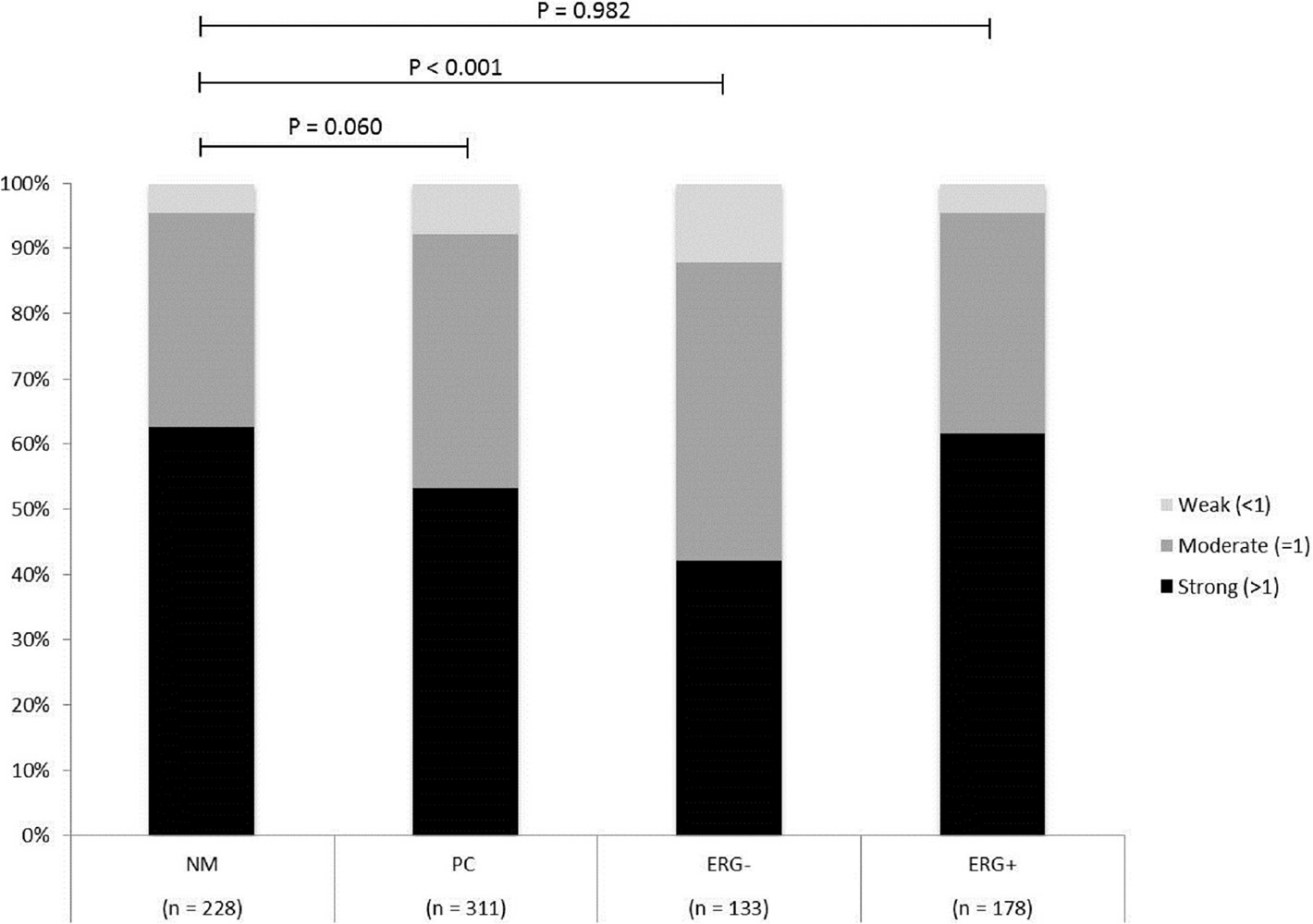
5hmC scores in PC and NM cores. Distribution of 5hmC scores in the full PC patient set and *ERG+* and *ERG−* PCs. For each patient, 5hmC scores were determined as the mean grade of minimum two PC or NM cores, respectively. *P* values from chi^2^ test

To systematically investigate 5hmC levels in NM and PC tissue samples, we analyzed a large RP tissue microarray by immunohistochemistry (clinical data in Table 1). Nuclear 5hmC staining intensity in prostate epithelial cells was evaluated and given a numerical grade (0, no staining; 1, moderate staining; 2, strong staining; Fig. 1a–d). A 5hmC score was then calculated as the mean grade of at least two malignant or 2 NM cores, respectively (5hmC score <1, weak; =1, moderate; >1, strong). Initially, we compared 5hmC scores for 311 patients for whom we could evaluate 5hmC staining in at least 2 malignant cores and 228 patients for whom 5hmC staining was assessable in 2 NM cores (Table 1). The vast majority (96 %) of NM samples displayed strong or intermediate 5hmC staining (Fig. 2). In the full patient set (*n* = 311), 5hmC levels were slightly reduced in PC compared to NM tissue samples (Fig. 1a), but the difference was only borderline significant (Fig. 2; *p* = 0.06, chi^2^ test). This finding is in agreement with results from two previous immunohistochemistry (IHC) studies that used relatively small PC patient sample sets [27, 37].

**Table 1:** Clinical data for PC patients represented on the radical prostatectomy tissue microarray

	546 RP patients included on TMA	311 RP patients for whom a 5hmC score could be evaluated in malignant cores	178 *ERG* positive RP patients for whom a 5hmC score could be evaluated in malignant cores	133 *ERG* negative RP patients for whom a 5hmC score could be evaluated in malignant cores
Age at RP, median (range)	64 (36–77)	63 (36–76)	63 (48–74)	63 (36–76)
Pathological Gleason score				
=6, *n* (%)	178 (32.6 %)	88 (28 %)	62 (35 %)	26 (20 %)
=7, *n* (%)	270 (49.5 %)	172 (55 %)	98 (55 %)	74 (56 %)
≥8, *n* (%)	98 (17.9 %)	51 (16 %)	18 (10 %)	33 (25 %)
Pathological T stage (*n*)				
≤pT2c, *n* (%)	363 (66.5 %)	205 (66 %)	116 (65 %)	89 (67 %)
≥pT3a, *n* (%)	182 (33.3 %)	105 (34 %)	62 (35 %)	43 (32 %)
Unknown, *n* (%)	1 (0.2 %)	1 (<1 %)	0 (0.0 %)	1 (1 %)
Pre-operative PSA				
PSA ≤10 ng/ml, *n* (%)	222 (40.7 %)	132 (42 %)	83 (47 %)	49 (37 %)
PSA >10 ng/ml, *n* (%)	324 (59.3 %)	179 (58 %)	95 (53 %)	84 (63 %)
Surgical margin status				
Negative, *n* (%)	174 (31.9 %)	211 (68 %)	122 (69 %)	89 (67 %)
Positive, *n* (%)	366 (67.0 %)	95 (31 %)	52 (29 %)	43 (32 %)
Unknown, *n* (%)	6 (1.1 %)	5 (2 %)	4 (2 %)	1 (1 %)
Lymph node status				
Positive, *n* (%)	0 (0.0 %)	0 (0.0 %)	0 (0.0 %)	0 (0.0 %)
Negative, n (%)	366 (67.0 %)	98 (32 %)	57 (32 %)	41 (31 %)
Unknown, *n* (%)	180 (33.0 %)	213 (68 %)	121 (68 %)	92 (69 %)
Median follow-up, months (range)	79.7 (0–157.5)	79.8 (12.2–157.5)	80.4 (12.3–157.5)	78.7 (12.2–140.9)
PSA recurrence, *n* (%)	236 (43.2 %)	143 (46 %)	83 (46.6 %)	60 (45 %)
No PSA recurrence, *n* (%)	310 (56.8 %)	168 (54 %)	95 (53.4 %)	73 (55 %)
*ERG* status (determined by IHC)				
Positive, *n* (%)	289 (52.9 %)	178 (57 %)	178 (100 %)	0 (0.0 %)
Negative, *n* (%)	242 (44.3 %)	133 (43 %)	0 (0.0 %)	133 (100 %)
Unknown, *n* (%)	14 (2.6 %)	0 (0.0 %)	0 (0.0 %)	0 (0.0 %)

Next, we investigated 5hmC levels in PCs stratified by *ERG* status, as determined by an IHC-based method that has >95 % sensitivity and specificity for detecting *ERG* rearrangements in PC [45, 46]. For *ERG−* PCs (*n* = 133, Table 1), malignant cores showed significantly less strong (42 vs*.* 63 %), more moderate (46 vs*.* 33 %), and more weak (12 vs*.* 4 %) 5hmC staining compared to NM cores (Fig. 2), a difference that was highly statistically significant (*p* < 0.001, chi^2^ test; Fig. 2). In contrast, *ERG+* PCs (*n* = 178, Table 1) displayed an almost identical score distribution as seen in NM cores (strong, 62 vs*.* 63 %; moderate, 34 vs*.* 33 %; weak, both 4 %; *p* = 0.98, chi^2^ test; Fig. 2). No difference was observed in 5hmC scores in NM tissue samples from patients with *ERG+* (*n* = 106) vs*. ERG−* (*n* = 122) PC (*p* = 0.18, chi^2^ test; data not shown). Together, these results indicate that loss of 5hmC in PC occurs preferentially in *ERG−* cases.

### Correlation between 5hmC levels and clinicopathological parameters in PC

Next, we investigated possible correlations between 5hmC score in malignant cores and routine clinicopathological parameters pre-operative PSA, GS, pathological tumor stage (pT), surgical margin (SM), and biochemical recurrence (BCR) status. In the full patient set, strong 5hmC staining (score >1) was significantly associated with BCR (*p* = 0.018, chi^2^ test), whereas no significant correlations were seen with any of the other parameters (*p* > 0.39, chi^2^ test; Additional file 1: Figure S1). Notably, by subgroup analysis, we found that strong 5hmC staining (score >1) was significantly associated with BCR in the *ERG−* (*p* = 0.006, chi^2^ test; Additional file 2: Figure S2) but not in the *ERG+* subset (*p* = 0.45; Additional file 3: Figure S3). Strong 5hmC staining was also significantly associated with advanced pT stage (pT3-4) in *ERG−* (*p* = 0.001, chi^2^ test; Additional file 2: Figure S2), but not in *ERG+* PCs, where instead a borderline significant trend towards reduced 5hmC staining in pT3-4 stage tumors was seen (*p* = 0.06, chi^2^ test; Additional file 3: Figure S3). There was no significant association between 5hmC score and pre-operative PSA, GS, or SM status in either the *ERG+* or *ERG−* subset (*p* > 0.12; Additional file 2: Figure S2, Additional file 3: Figure S3). In summary, these findings suggest that high 5hmC levels may be associated with tumor progression in *ERG−* PC.

### Prognostic value of 5hmC levels in PC

**Fig. 3:**
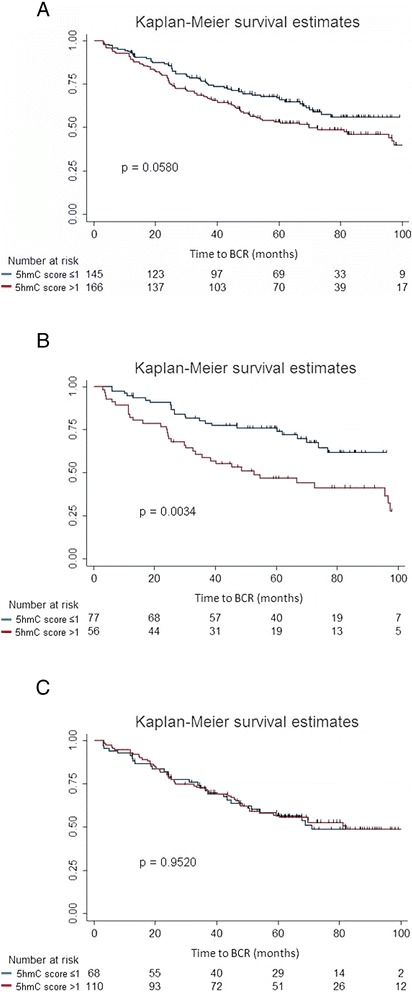
Kaplan-Meier analysis: Association between 5hmC score and time to BCR after RP. **a** High 5hmC score (>1) was a borderline significant predictor of time to BCR in the full PC patient set (*n* = 311; *p* = 0.058, log-rank test). **b** High (>1) 5hmC score was a significant adverse predictor of time to BCR in *ERG−* PC (*n* = 133; *p* = 0.003, log-rank test). **c** 5hmC score did not predict time to BCR in *ERG+* PC (*n* = 178; *p* = 0.95, log-rank test)

To assess the potential prognostic value of 5hmC levels in PC, we investigated whether 5hmC score in malignant cores was associated with time to BCR after RP. In the full patient set (*n* = 311), a high 5hmC score (analyzed as a continuous variable) was significantly associated with shorter time to BCR in univariate Cox regression analysis (hazard ratio (HR) (95 % confidence interval (CI)): 1.53 (1.09–2.15), *p* = 0.013, Table 2). Likewise, high GS, advanced pT stage, high pre-operative PSA, and positive SM status predicted early BCR in univariate analysis (*p* < 0.001, Table 2), whereas *ERG* status had no prognostic value in this patient set (*p* = 0.73, Table 2). Notably, 5hmC score remained significant also in multivariate Cox regression analysis (HR (95 % CI): 1.62 (1.15–2.28), *p* = 0.006, Table 2) together with all routine clinicopathological parameters (*p* ≤ 0.003, Table 2). We used Harrell’s C-index to estimate predictive accuracies of the multivariate models, which remained at 0.75 whether 5hmC score was included or not (Table 2). When 5hmC score was analyzed as a dichotomized variable (≤1 vs*.* >1) in the full patient set, it was only borderline significant in uni- and multivariate cox regression analysis (*p* = 0.059 and *p* = 0.066, respectively; Additional file 4: Table S1) and in Kaplan-Meier analysis (*p* = 0.058; Fig. 3a)

**Table 2:** Uni-and multivariate Cox regression analysis of BCR in the full PC patient set

	All PCs (*n* = 311, 143 BCR)								
	Univariate			Multivariate^a^		Multivariate^b^			
Variable	HR (95 % CI)	*p* value	C-index	HR (95 % CI)	*p* value	HR (95 % CI)	*p* value	C-index^c^	C-index^d^
5hmC score (cont.)	1.53 (1.09–2.15)	**0.013**	0.57	1.58 (1.12–2.23)	**0.009**	1.62 (1.15–2.28)	**0.006**	0.75	
Pre-op. PSA (≤10 vs*.* >10 ng/ml)	2.93 (2.00–4.29)	**<0.001**	0.63	2.17 (1.46–3.24)	**<0.001**	2.17 (1.46–3.24)	**<0.001**		0.75
Surgical margin (neg. vs*.* pos.)	2.86 (2.04–4.00)	**<0.001**	0.63	1.99 (1.38–2.86)	**<0.001**	1.99 (1.38–2.86)	**<0.001**		
Tumor stage (pT2 vs*.* pT3-4)	2.96 (2.13–4.13)	**<0.001**	0.64	1.91 (1.32–2.76)	**0.001**	1.90 (1.32–2.75)	**0.001**		
Gleason score (≤6 vs*.* >6)	2.67 (1.70–4.17)	**<0.001**	0.58	2.08 (1.30–3.34)	**<0.002**	2.02 (1.26–3.23)	**0.003**		
*ERG* status (neg. vs*.* pos.)	1.06 (0.76–1.48)	0.732	0.51	1.19 (0.85–1.68)	0.314	–	–		

Significant *p* values (*p* < 0.05) are highlighted in bold

^a^Global multivariate model including all parameters

^b^Final multivariate model including significant variables only

^c^Harrell’s C-index for final model including 5hmC

^d^Harrell’s C-index for final model excluding 5hmC

Recurrence-free survival analyses were also performed after patient stratification by *ERG* status. Pre-operative PSA, GS, pT stage, and SM status were significant predictors of time to BCR in univariate analysis in both patient subgroups (*p* < 0.05, Table 3), indicating that this is a representative RP cohort. Furthermore, a high 5hmC score (analyzed as a continuous variable) was a significant adverse predictor of BCR in the *ERG−* subgroup in univariate (HR (95 % CI), 1.97 (1.19–3.26), *p* = 0.008) as well as multivariate Cox regression analysis (HR (95 % CI), 2.02 (1.16–3.51), *p* = 0.013), whereas no significant association was found for *ERG+* PCs (Table 3). For the *ERG−* PC subgroup, addition of 5hmC score to a multivariate model containing clinicopathological factors only (pre-operative PSA and SM status) improved Harrell’s C-index from 0.68 to 0.73, suggesting moderately improved predictive accuracy. Pathological T stage and GS were excluded from the final model because they failed in multivariate analysis (Table 3). Similar results were obtained when 5hmC score was analyzed as a dichotomized variable (≤1 vs*.* >1) in uni- and multivariate Cox regression analysis (Additional file 5: Table S2). A high 5hmC score (>1) was also significantly associated with shorter time to BCR in Kaplan-Meier analysis in *ERG−* PCs (*p* = 0.003, log-rank test; Fig. 3b), whereas no difference was observed between the high/low 5hmC score subgroups for *ERG+* PCs (*p* = 0.95, log-rank test; Fig. 3c).

**Table 3:** Uni-and multivariate Cox regression analysis of BCR in *ERG* stratified PC patient subsets

	*ERG−* (*n* = 133, 60 BCR)								
	Univariate			Multivariate^a^		Multivariate^b^			
Variable	HR (95 % CI)	*p* value	C-index	HR (95 % CI)	*p* value	HR (95 % CI)	*p* value	C-index^c^	C-index^d^
5hmC score (cont.)	1.97 (1.19–3.26)	**0.008**	0.62	2.02 (1.16–3.51)	**0.013**	2.08 (1.22–3.52)	**0.007**	0.73	
Pre-op. PSA (≤10 vs*.* >10 ng/ml)	2.66 (1.44–4.92)	**0.002**	0.60	2.36 (1.26–4.44)	**0.008**	2.62 (1.41–4.87)	**0.002**		0.68
Surgical margin (neg. vs*.* pos.)	2.83 (1.69–4.72)	**<0.001**	0.62	2.51 (1.42–4.44)	**0.001**	2.80 (1.67–4.68)	**<0.001**		
Tumor stage (pT2 vs*.* pT3-4)	2.26 (1.35–3.78)	**0.002**	0.62	1.35 (0.74–2.45)	0.328	–	–		
Gleason score (<7 vs*.* ≥7)	2.22 (1.01–4.88)	**0.048**	0.56	1.82 (0.81–4.10)	0.147	–	–		
	*ERG+* (*n* = 178, 83 BCR)								
	Univariate			Multivariate^a^		Multivariate^b^			
Variable	HR (95 % CI)	*p* value	C-index	HR (95 % CI)	*p* value	HR (95 % CI)	*p* value	C-index^c^	C-index^d^
5hmC score (cont.)	1.22 (0.77–1.94)	0.398	0.52	1.44 (0.90–2.30)	0.129	–	–	NA	
Pre-op. PSA (≤10 vs*.* >10 ng/ml)	3.19 (1.96–5.20)	**<0.001**	0.65	1.93 (1.12–3.32)	**0.017**	2.03 (1.26–3.25)	**0.004**		0.76
Surgical margin (neg. vs*.* pos.)	2.91 (1.86–4.54)	**<0.001**	0.63	1.73 (1.07–2.82)	**0.026**	1.94 (1.25–3.01)	**0.003**		
Tumor stage (pT2 vs*.* pT3-4)	3.65 (2.35–5.68)	**<0.001**	0.65	2.35 (1.41–3.91)	**0.001**	2.13 (1.36–3.35)	**0.001**		
Gleason score (<7 vs*.* ≥7)	3.04 (1.76–5.27)	**<0.001**	0.61	2.23 (1.24–4.01)	**0.007**	2.76 (1.63–4.68)	**<0.001**		

Significant *p* values (*p* < 0.05) are highlighted in bold

*NA* not applicable.

^a^Global multivariate model including all parameters

^b^Final multivariate model including significant variables only

^c^Harrell’s C-index for final model including 5hmC

^d^Harrell’s C-index for final model excluding 5hmC

Finally, we note that despite the significant association between high 5hmC score and advanced pT stage in *ERG−* PCs (Additional file 2: Figure S2), both 5hmC score and pT stage remained significant predictors of time to BCR in a bivariate Cox regression model (5hmC score; HR (95 % CI), 1.67 (1.01–2.81), *p* = 0.044; pT stage, HR (95 % CI), 1.96 (1.14–3.35), *p* = 0.014). The C-index for this model was 0.66, i.e., higher than for either parameter alone (Table 3). We also performed Kaplan-Meier analysis separately for pT2 and pT3-4 stage *ERG−* PCs, respectively. A high 5hmC score (≥1) was significantly associated with BCR in the pT2 stage subgroup (*p* = 0.029, log-rank test, Additional file 6: Figure S4a) and a similar trend was observed for the pT3-4 subgroup, although statistical significance was not reached in this smaller patient subset (*p* = 0.35, log-rank test, Additional file 6: Figure S4b). In conclusion, our results indicate that a high 5hmC level is a significant adverse predictor of time to BCR after RP in patients with *ERG−* PC, independent of routine clinicopathological variables.

## Discussion

The present study is the first large-scale investigation of 5hmC levels in PC, as well as the first study to explore and demonstrate a prognostic potential for 5hmC in this common malignancy. By immunohistochemical analysis of a large RP cohort, we found that 5hmC levels were significantly reduced in *ERG−* but not in *ERG+* PC as compared to NM prostate tissue samples. Furthermore, we identified 5hmC as a significant predictor of biochemical recurrence in *ERG−* PC, independent of routine clinicopathological factors. In contrast, 5hmC had no prognostic value in *ERG+* PCs. *ERG* status was also not associated with BCR in this RP patient cohort, consistent with previous reports [5].

Our findings confirm and expand on results from two previous studies [27, 37] that reported loss of 5hmC immunoreactivity in PC, but based on analysis of only 30 PC vs*.* 10 NM and 11 PC vs*.* 11 NM tissue samples, respectively. The prognostic potential of 5hmC was not explored in these studies, nor was the association between 5hmC and *ERG* status. The small-scale study design also prohibited an evaluation of 5hmC levels in relation to routine clinicopathological parameters in these earlier reports [27, 37]. Correlations between reduced 5hmC levels and mutations in 5hmC regulating enzymes, such as *TET*s, have been identified in various other human malignancies [29, 30, 34, 35], but such mutations are rare in PC [47, 48]. Thus, further studies are needed to investigate how 5hmC levels are regulated in normal as well as in *ERG−* and *ERG+* PC cells.

Our results highlight the importance of *ERG* stratification in PC biomarker studies. Although not understood in detail, the molecular pathways that operate in *ERG+* tumors are relatively better described than the mechanisms involved in development/progression of *ERG−* PCs [11]. It is becoming increasingly clear that *ERG+* and *ERG−* PCs represent distinct molecular subtypes and that subtype-specific markers may be required for prediction of tumor aggressiveness [14]. Thus, similar to our findings for 5hmC, previous studies have shown that the prognostic potential of certain PC biomarker candidates is dependent on *ERG* status, e.g., *SPINK1* and *SPOP* as well as some DNA methylation marker candidates [6–12].

It is intriguing that while we observed a general loss of 5hmC in *ERG−* PCs, our results indicated that retaining a high global level of 5hmC is associated with *ERG−* PC progression. Interestingly, hypoxia has previously been associated with poor prognosis after RP [49] and, together with reactive oxygen species (ROS), has been shown to induce *TET1* expression, leading to activation of hypoxia-inducible genes through conversion of 5mC to 5hmC in both a global and site-specific manner [50–52]. While we did not find significant differential expression of *TET1* or common hypoxia response genes (*HIF1A*, *HIF2A*, *CA9*, *PGK1*, *GPI*, *VEGFA*, *BNIP*, *ENO1*) [50–52] in *ERG+* vs*. ERG−* PCs in publicly available datasets [53–55] (data not shown), further studies are warranted to investigate whether high levels of hypoxia may exist in a subset of *ERG−* PC and thus potentially could be linked to elevated 5hmC levels and poor prognosis.

In line with our finding that high levels of 5hmC were associated with early BCR in *ERG−* PC, high levels of 5hmC have also been identified as an independent predictor of poor prognosis in AML [40]. Furthermore, the same study reported highly variable 5hmC levels between different AML samples [40], consistent with our observations for both *ERG−* and *ERG+* PCs. Likewise, 5hmC levels have been shown to vary between different types of brain cancer, with oligodendroglial tumors displaying overall high 5hmC levels, while reduced 5hmC levels were found in adult glioblastoma and anaplastic astrocytoma and associated with poor prognosis [29]. Loss of 5hmC has also been reported to predict adverse outcome in melanoma [34], myelodysplastic syndromes [35], and gastric cancer [38]. Together, results from these previous reports and our current results for 5hmC in PC indicate that potential prognostic implications of 5hmC are cancer (sub)type-specific, which is also in accordance with the highly tissue-specific distribution of 5hmC in normal cells [33, 43].

There are some limitations to the present study. First, the prognostic value of 5hmC in *ERG−* but not in *ERG+* PC needs further validation in large independent patient cohorts. Moreover, we used BCR as endpoint, which is only a surrogate for tumor aggressiveness. Thus, the potential prognostic value of 5hmC should also be assessed in relation to more clinically relevant endpoints, such as metastatic progression or PC-specific and overall mortality. Due to the slowly progressing nature of PC, analysis of such endpoints would require at least 15 years of follow-up [2].

Also limiting our study is the lack of 5mC data for our sample set; thus, we have not investigated the correlation between 5mC and 5hmC levels. However, previous studies have reported no clear correlation between these two epigenetic marks [27, 43]. Furthermore, we have not investigated the expression levels of enzymes involved in 5hmC regulation, such as TETs or isocitrate dehydrogenases (IDHs) [48], which should be investigated in future studies.

Another potential limitation of our study is the use of ERG immunoreactivity as a proxy for *ERG* gene fusion status. However, previous studies have demonstrated that ERG immunoreactivity is highly concordant (>95 %) with *ERG* rearrangement status in PC tissue samples [45, 46]. Moreover, ERG overexpression is considered a PC “driver” event whether caused by translocation, copy number alteration, or other mechanisms [56]. Furthermore, while *ERG* status may differ between distinct cancer foci in multifocal PC [57], we are confident of the match between 5hmC score and *ERG* status in the present study, as these were assessed on consecutive sections of the exact same cores. Finally, although this is the first large-scale evaluation of 5hmC in PC, immunohistochemical analysis is semi-quantitative and only allows assessment of global 5hmC levels. Our results warrant further studies of the genomic distribution of 5hmC in ERG− vs*.* ERG+ PC as well as in NM prostate tissue samples. A number of recently developed NGS-based protocols allow quantitative genome wide or whole-genome profiling of the 5hmC methylome [58], which could be used to increase our understanding of the epigenetic mechanisms involved in PC development and progression.

## Conclusions

This is the first large-scale study of 5hmC in PC as well as the first study to demonstrate a prognostic potential for 5hmC in prostate adenocarcinoma. In conclusion, we found that the global level of 5hmC was significantly reduced in *ERG−* but not in *ERG+* PC, as compared to NM prostate tissue samples. Furthermore, we found that a subgroup of *ERG−* tumors retained a high 5hmC level that was associated with early BCR after RP, whereas 5hmC had no significant prognostic value in *ERG+* PC in our patient set. Our results highlight the importance of *ERG* stratification in PC biomarker studies and suggest that epigenetic mechanisms involving 5hmC are important in *ERG−* PC tumorigenesis and development. Future studies, using large independent PC patient cohorts, are needed to confirm our findings. Finally, in order to better understand the role of epigenetic changes in PC development and progression, it will be important to generate genome wide maps of the 5-hydroxymethylome in malignant and NM tissue samples, as well as in specimens from aggressive and non-aggressive PC, while taking *ERG* status into account. Such future studies could aid in the identification of important genes and pathways involved in PC development and progression, providing a deeper understanding of epigenomic reprogramming in PC.

## Availability of supporting data

The data sets supporting the results of this article are included within the article and its additional files.

## Methods

### Radical prostatectomy TMA

A tissue microarray (TMA) was generated using formalin-fixed paraffin-embedded tissue samples from 552 radical prostatectomies of histological verified clinically localized PC, surgically removed with curative intent between 1998 and 2009 at Department of Urology, Aarhus University Hospital, Denmark. In all cases, Gleason scores were reassigned according to International Union Against Cancer and WHO/International Society of Urological Pathology criteria [59]. For each patient, a trained pathologist with extensive experience in prostate histopathology (SH) identified a representative malignant tissue area in a hematoxylin and eosin-stained section from the original prostatectomy specimens. Likewise, a representative area of adjacent NM prostate tissue was selected in parallel for a subset of the patients (*n* = 301, chosen at random). For each patient, three cores (1 mm diameter) were punched from the selected malignant tissue area and two cores were punched from the NM tissue area. The cores were mounted in a total of 16 individual TMA blocks using the TMA master (3DHISTECH, Hungary).

The most recent clinical follow-up of time to PSA recurrence after RP was conducted in May 2015 for all 552 patients on the TMA. At this time, 6 patients had withdrawn consent, while another 101 patients were excluded due to either pre- or post-operative endocrine or radiation treatment, short (<3 months) follow-up, or BCR within 3 months from RP (Additional file 7: Figure S5). Clinical data for all patients included on the TMA, except for the six who withdrew consent, is listed in Table 1.

This study was approved by the regional ethical committee and the Danish Data Protection Agency. All patients provided written informed consent.

### IHC

#### 5hmC staining and evaluation

TMA tissue sections (4 μm) were deparaffinized (Tissue-Tek Tissue-Clear Xylene Substitute, Sakura) and re-hydrated according to standard protocols and antigen retrieval was carried out in two subsequent steps, as described previously [27]. Briefly, heat induced epitope retrieval with citrate buffer (pH 6.0) was performed, followed by incubation in 3.5 N HCl for 15 min at room temperature. The subsequent procedure was performed on the Autostainer Link48 (DAKO, Denmark) at room temperature: rabbit polyclonal 5-hmC specific antibody (Active Motif, Carlsbad, CA, cat. no 39769) was applied at a 1:1000 dilution and incubated for 60 min. Horse radish peroxidase (HRP) conjugated rabbit secondary antibody (Envision) was applied for 30 min. TMA sections were subsequently counterstained with hematoxylin. As a negative control, the primary antibody was omitted. This primary antibody and IHC staining protocol has been shown to be highly 5hmC specific in multiple previously published reports [21, 27–29, 37, 38, 41–44], including several antigen competition experiments, thus proving the sensitivity and specificity of this antibody for 5hmC.

For each core on the TMA, 5hmC immunoreactivity was scored individually by two independent observers (SH and SHS) using the Pannoramic Viewer software (3DHISTECH, Hungary). In cases of disagreement, cores were reassessed to reach a consensus score. Kappa statistics showed very high inter-observer agreement (Kappa index = 0.95; *p* < 0.0001). Nuclear 5hmC staining intensity in malignant or NM prostate epithelial cells was evaluated and given a numerical grade (0, no staining; 1, moderate staining; 2, strong staining). Due to progressive TMA slicing (sections used for other projects), some cores had changed status from malignant to NM from the time of TMA construction. Thus, for 70 patients, all malignant cores were lost during TMA processing and 64 patients had less than two malignant cores that could be evaluated for 5hmC staining. Patients for whom we could not evaluate 5hmC staining intensity in at least two NM or at least two PC cores were excluded from further analysis. Then, for each patient, a 5hmC score was calculated as the mean grade of at least two malignant cores or two NM cores, respectively (score <1, weak; 1, moderate; >1, strong). In total, a 5hmC score was calculated in malignant tissue from 311 patients (Additional file 7: Figure S5) and a 5hmC score was calculated in NM tissue from 228 patients (Additional file 8: Figure S6).

#### ERG staining

TMA tissue sections (2.5 μm) were deparaffinized and epitopes were retrieved using TEG buffer, as previously described [60]. TMA sections were stained with rabbit monoclonal *ERG* antibody (2805–1, Epitomics) in a 1:150 dilution in TEG. Secondary staining was performed using the EnVision + System (HRP Labelled Polymer Anti-Rabbit K4003, DakoCytomation). For all cores, ERG immunoreactivity (0,no staining; 1, moderate; 2, strong) was evaluated and scored by two independent observers (SH and ASL) as described above. Positive staining was used as a proxy for *ERG* fusion status as previously described [45, 46].

### Statistics

All statistical analyses were performed using STATA v. 11.2 (StataCorp, College Station TX, USA). In all cases, *p* < 0.05 was considered significant. Associations between 5hmC score and clinicopathological variables were assessed by the chi^2^ test. Biochemical recurrence (BCR), defined as PSA >0.2 ng/ml on two consecutive measurements, was used as the clinical endpoint in univariate and multivariate Cox regression analyses as well as in Kaplan-Meier analyses. Patients without BCR were censored at their last normal PSA measurement. Statistical significance in Kaplan-Meier analysis was evaluated using two-sided log-rank tests. Predictive accuracy was estimated using Harrell’s C-index.

## Additional files

Additional file 1: Figure S1. Distribution of 5hmC scores by clinicopathological parameters and BCR in the full PC patient set. Full PC patient set, *n* = 311. *P* values: chi^2^ test. (TIFF 145 kb) Additional file 2: Figure S2. Distribution of 5hmC scores by clinicopathological parameters and BCR status in *ERG−* PC. *ERG−* subset: *n* = 133. *P* values: chi^2^ test. (TIFF 146 kb) Additional file 3: Figure S3. Distribution of 5hmC scores by clinicopathological parameters and BCR status in *ERG+* PC. *ERG+* subset: *n* = 178. *P* values: chi^2^ test. (TIFF 143 kb) Additional file 4: Table S1. Uni- and multivariate Cox regression analysis of BCR after RP in the full PC patient set. 5hmC analyzed as a dichotomized variable. (DOCX 33 kb) Additional file 5: Table S2. Uni- and multivariate Cox regression analysis of BCR after RP in *ERG−/ERG+* PC patients. 5hmC analyzed as a dichotomized variable. ^a^ Global multivariate model including all parameters. ^b^ Final multivariate model including significant variables only. ^c^ Harrell’s C-index for final model including 5hmC. ^d^ Harrell’s C-index for final model excluding 5hmC. NA: not applicable. Significant *p* values (*p* < 0.05) are highlighted in bold. (DOCX 36 kb) Additional file 6: Figure S4. Kaplan-Meier analysis: 5hmC score and time to BCR in *ERG−* PC stratified by pT stage. A: High 5hmC score (>1) was a significant adverse predictor of time to BCR in pT2 stage *ERG−* PCs (*p* = 0.029, log-rank test). B: The same trend was seen in pT3-4 stage *ERG−* PCs, but this was not statistically significant (*p* = 0.35, log-rank test). (TIFF 183 kb) Additional file 7: Figure S5. Flow chart illustrating the sample inclusion/exclusion process (malignant cores). A total of 552 PC patients were represented by malignant cores on the TMA. The final sample set used for all subsequent analyses consisted of 5hmC scores from 311 PC patients for whom at least 2 malignant cores could be evaluated for 5hmC staining intensity (grade). (TIFF 68 kb) Additional file 8: Figure S6. Flow chart illustrating the sample inclusion/exclusion process (NM cores). A total of 301 PC patients were represented by NM cores on the TMA. The final sample set used for all subsequent analyses consisted of 5hmC scores from 228 PC patients for whom at least 2 NM cores could be evaluated for 5hmC staining intensity (grade). (TIFF 58 kb)

## Abbreviations

5hmC: 5-hydroxymethylcytosine
5mC: 5-methylcytosine
AML: acute myeloid leukemia
BCR: biochemical recurrence
CI: confidence interval
GS: Gleason score
HR: hazard ratio
IHC: immunohistochemistry
NM: non-malignant
PC: prostate cancer
PSA: prostate-specific antigen
pT: pathological tumor stage
RP: radical prostatectomy
SM: surgical margin
TMA: tissue microarray

## References

[1] International Agency for Research on Cancer, W.H.O. Globocan 2012. http://globocan.iarc.fr/Pages/fact_sheets_cancer.aspx. Accessed on May 14, 2015.

[2] Albertsen PC, Hanley JA, Fine J (2005). 20-year outcomes following conservative management of clinically localized prostate cancer. JAMA.

[3] Telesca D, Etzioni R, Gulati R (2008). Estimating lead time and overdiagnosis associated with PSA screening from prostate cancer incidence trends. Biometrics.

[4] Tomlins SA, Rhodes DR, Perner S, Dhanasekaran SM, Mehra R, Sun XW (2005). Recurrent fusion of TMPRSS2 and ETS transcription factor genes in prostate cancer. Science.

[5] Bostrom, P.J.; Bjartell, A.S.; Catto, J.W.; Eggener, S.E.; Lilja, H.; Loeb, S.; Schalken, J.; Schlomm, T.; Cooperberg, M.R. Genomic predictors of outcome in prostate cancer. *European urology* 2015: doi: 10.1016/j.eururo.2015.04.008 10.1016/j.eururo.2015.04.008 25913390

[6] Barbieri CE, Baca SC, Lawrence MS, Demichelis F, Blattner M, Theurillat JP (2012). Exome sequencing identifies recurrent SPOP, FOXA1 and MED12 mutations in prostate cancer. Nat Genet.

[7] Brase JC, Johannes M, Mannsperger H, Falth M, Metzger J, Kacprzyk LA (2011). TMPRSS2-ERG-specific transcriptional modulation is associated with prostate cancer biomarkers and TGF-beta signaling. BMC Cancer.

[8] Gasi Tandefelt D, Boormans JL, van der Korput HA, Jenster GW, Trapman J (2013). A 36-gene signature predicts clinical progression in a subgroup of ERG-positive prostate cancers. Eur Urol.

[9] Karnes RJ, Cheville JC, Ida CM, Sebo TJ, Nair AA, Tang H (2010). The ability of biomarkers to predict systemic progression in men with high-risk prostate cancer treated surgically is dependent on ERG status. Cancer Res.

[10] Kron K, Liu L, Trudel D, Pethe V, Trachtenberg J, Fleshner N (2012). Correlation of ERG expression and DNA methylation biomarkers with adverse clinicopathologic features of prostate cancer. Clin Cancer Res.

[11] Tomlins SA, Rhodes DR, Yu J, Varambally S, Mehra R, Perner S (2008). The role of SPINK1 in ETS rearrangement-negative prostate cancers. Cancer Cell.

[12] Vinarskaja A, Schulz WA, Ingenwerth M, Hader C, Arsov C (2013). Association of PITX2 mRNA down-regulation in prostate cancer with promoter hypermethylation and poor prognosis. Urol Oncol.

[13] Kron K, Trudel D, Pethe V, Briollais L, Fleshner N, van der Kwast T (2013). Altered DNA methylation landscapes of polycomb-repressed loci are associated with prostate cancer progression and ERG oncogene expression in prostate cancer. Clin Cancer Res.

[14] Tomlins SA, Alshalalfa M, Davicioni E, Erho N, Yousefi K, Zhao S (2015). Characterization of 1577 primary prostate cancers reveals novel biological and clinicopathologic insights into molecular subtypes. Eur Urol.

[15] Dobosy JR, Roberts JL, Fu VX, Jarrard DF (2007). The expanding role of epigenetics in the development, diagnosis and treatment of prostate cancer and benign prostatic hyperplasia. J Urol.

[16] Chiam K, Ricciardelli C, Bianco-Miotto T (2014). Epigenetic biomarkers in prostate cancer: current and future uses. Cancer Lett.

[17] Haldrup C, Mundbjerg K, Vestergaard EM, Lamy P, Wild P, Schulz WA (2013). DNA methylation signatures for prediction of biochemical recurrence after radical prostatectomy of clinically localized prostate cancer. J Clin Oncol.

[18] Kristensen H, Haldrup C, Strand S, Mundbjerg K, Mortensen MM, Thorsen K (2014). Hypermethylation of the GABRE~mir-452~mir-224 promoter in prostate cancer predicts biochemical recurrence after radical prostatectomy. Clin Cancer Res.

[19] Strand SH, Orntoft TF, Sorensen KD (2014). Prognostic DNA methylation markers for prostate cancer. Int J Mol Sci.

[20] Tahiliani M, Koh KP, Shen Y, Pastor WA, Bandukwala H, Brudno Y (2009). Conversion of 5-methylcytosine to 5-hydroxymethylcytosine in mammalian DNA by MLL partner TET1. Science.

[21] Ito S, D'Alessio AC, Taranova OV, Hong K, Sowers LC, Zhang Y (2010). Role of Tet proteins in 5mc to 5hmC conversion, ES-cell self-renewal and inner cell mass specification. Nature.

[22] Ito S, Shen L, Dai Q, Wu SC, Collins LB, Swenberg JA (2011). Tet proteins can convert 5-methylcytosine to 5-formylcytosine and 5-carboxylcytosine. Science.

[23] He YF, Li BZ, Li Z, Liu P, Wang Y, Tang Q (2011). Tet-mediated formation of 5-carboxylcytosine and its excision by TDG in mammalian DNA. Science.

[24] Ye C, Li L. 5-hydroxymethylcytosine: a new insight into epigenetics in cancer. Cancer Biol Ther. 2014;15:10–5. 10.4161/cbt.27144 PMC3938512 24253310

[25] Iurlaro M, Ficz G, Oxley D, Raiber EA, Bachman M, Booth MJ (2013). A screen for hydroxymethylcytosine and formylcytosine binding proteins suggests functions in transcription and chromatin regulation. Genome Biol.

[26] Huang Y, Pastor WA, Shen Y, Tahiliani M, Liu DR, Rao A (2010). The behaviour of 5-hydroxymethylcytosine in bisulfite sequencing. PLoS One.

[27] Haffner MC, Chaux A, Meeker AK, Esopi DM, Gerber J, Pellakuru LG (2011). Global 5-hydroxymethylcytosine content is significantly reduced in tissue stem/progenitor cell compartments and in human cancers. Oncotarget.

[28] Haffner MC, Pellakuru LG, Ghosh S, Lotan TL, Nelson WG, De Marzo AM, et al. Tight correlation of 5-hydroxymethylcytosine and polycomb marks in health and disease. Cell Cycle. 2013;12:1835–41. 10.4161/cc.25010 PMC3735697 23676216

[29] Orr BA, Haffner MC, Nelson WG, Yegnasubramanian S, Eberhart CG (2012). Decreased 5-hydroxymethylcytosine is associated with neural progenitor phenotype in normal brain and shorter survival in malignant glioma. PLoS One.

[30] Ko M, Huang Y, Jankowska AM, Pape UJ, Tahiliani M, Bandukwala HS (2010). Impaired hydroxylation of 5-methylcytosine in myeloid cancers with mutant TET2. Nature.

[31] Kraus TF, Globisch D, Wagner M, Eigenbrod S, Widmann D, Munzel M (2012). Low values of 5-hydroxymethylcytosine (5hmc), the "sixth base," are associated with anaplasia in human brain tumors. Int J Cancer.

[32] Kudo Y, Tateishi K, Yamamoto K, Yamamoto S, Asaoka Y, Ijichi H (2012). Loss of 5-hydroxymethylcytosine is accompanied with malignant cellular transformation. Cancer Sci.

[33] Li W, Liu M (2011). Distribution of 5-hydroxymethylcytosine in different human tissues. J Nucleic Acids.

[34] Lian CG, Xu Y, Ceol C, Wu F, Larson A, Dresser K (2012). Loss of 5-hydroxymethylcytosine is an epigenetic hallmark of melanoma. Cell.

[35] Liu X, Zhang G, Yi Y, Xiao L, Pei M, Liu S (2013). Decreased 5-hydroxymethylcytosine levels are associated with TET2 mutation and unfavorable overall survival in myelodysplastic syndromes. Leuk Lymphoma.

[36] Uribe-Lewis S, Stark R, Carroll T, Dunning MJ, Bachman M, Ito Y (2015). 5-hydroxymethylcytosine marks promoters in colon that resist DNA hypermethylation in cancer. Genome Biol.

[37] Yang H, Liu Y, Bai F, Zhang JY, Ma SH, Liu J (2013). Tumor development is associated with decrease of TET gene expression and 5-methylcytosine hydroxylation. Oncogene.

[38] Yang Q, Wu K, Ji M, Jin W, He N, Shi B (2013). Decreased 5-hydroxymethylcytosine (5-hmC) is an independent poor prognostic factor in gastric cancer patients. J Biomed Nanotechnol.

[39] Jin SG, Jiang Y, Qiu R, Rauch TA, Wang Y, Schackert G (2011). 5-Hydroxymethylcytosine is strongly depleted in human cancers but its levels do not correlate with IDH1 mutations. Cancer Res.

[40] Kroeze LI, Aslanyan MG, van Rooij A, Koorenhof-Scheele TN, Massop M, Carell T (2014). Characterization of acute myeloid leukemia based on levels of global hydroxymethylation. Blood.

[41] Globisch D, Munzel M, Muller M, Michalakis S, Wagner M, Koch S (2010). Tissue distribution of 5-hydroxymethylcytosine and search for active demethylation intermediates. PLoS One.

[42] Matsuda I, Imai Y, Hirota S (2014). Distinct global DNA methylation status in B-cell lymphomas: immunohistochemical study of 5-methylcytosine and 5-hydroxymethylcytosine. J Clin Exp Hematop.

[43] Nestor CE, Ottaviano R, Reddington J, Sproul D, Reinhardt D, Dunican D (2012). Tissue type is a major modifier of the 5-hydroxymethylcytosine content of human genes. Genome Res.

[44] Nettersheim D, Heukamp LC, Fronhoffs F, Grewe MJ, Haas N, Waha A (2013). Analysis of TET expression/activity and 5mc oxidation during normal and malignant germ cell development. PLoS One.

[45] Park K, Tomlins SA, Mudaliar KM, Chiu YL, Esgueva R, Mehra R (2010). Antibody-based detection of erg rearrangement-positive prostate cancer. Neoplasia.

[46] Braun M, Goltz D, Shaikhibrahim Z, Vogel W, Bohm D, Scheble V (2012). ERG protein expression and genomic rearrangement status in primary and metastatic prostate cancer—a comparative study of two monoclonal antibodies. Prostate Cancer Prostatic Dis.

[47] Kang MR, Kim MS, Oh JE, Kim YR, Song SY, Seo SI (2009). Mutational analysis of IDH1 codon 132 in glioblastomas and other common cancers. Int J Cancer.

[48] Kroeze LI, van der Reijden BA, Jansen JH (1855). 5-hydroxymethylcytosine: an epigenetic mark frequently deregulated in cancer. Biochim Biophys Acta.

[49] Vergis R, Corbishley CM, Norman AR, Bartlett J, Jhavar S, Borre M (2008). Intrinsic markers of tumour hypoxia and angiogenesis in localised prostate cancer and outcome of radical treatment: a retrospective analysis of two randomised radiotherapy trials and one surgical cohort study. Lancet Oncol.

[50] Kang KA, Piao MJ, Kim KC, Kang HK, Chang WY, Park IC (2014). Epigenetic modification of Nrf2 in 5-fluorouracil-resistant colon cancer cells: involvement of TET-dependent DNA demethylation. Cell Death Dis.

[51] Mariani CJ, Vasanthakumar A, Madzo J, Yesilkanal A, Bhagat T, Yu Y (2014). TET1-mediated hydroxymethylation facilitates hypoxic gene induction in neuroblastoma. Cell Rep.

[52] Tsai YP, Chen HF, Chen SY, Cheng WC, Wang HW, Shen ZJ (2014). TET1 regulates hypoxia-induced epithelial-mesenchymal transition by acting as a co-activator. Genome Biol.

[53] Zhu Y, Qiu P, Ji Y (2014). TCGA-assembler: open-source software for retrieving and processing TCGA data. Nat Methods.

[54] Zhu Y, Xu Y, Helseth Jr DL, Gulukota K, Yang S, Pesce LL, et al. Zodiac: a comprehensive depiction of genetic interactions in cancer by integrating TCGA data. J Natl Cancer Inst. 2015;107. 10.1093/jnci/djv129 PMC4554190 25956356

[55] Long Q, Xu J, Osunkoya AO, Sannigrahi S, Johnson BA, Zhou W (2014). Global transcriptome analysis of formalin-fixed prostate cancer specimens identifies biomarkers of disease recurrence. Cancer Res.

[56] Adamo P, Ladomery MR. The oncogene ERG: a key factor in prostate cancer. Oncogene. 2015. 10.1038/onc.2015.109 25915839

[57] Burdova A, Bouchal J, Tavandzis S, Kolar Z (2014). TMPRSS2-ERG gene fusion in prostate cancer. Biomed Pap Med Fac Univ Palacky Olomouc Czechoslovakia.

[58] Yardimci H, Zhang Y (2015). Charting oxidized methylcytosines at base resolution. Nat Struct Mol Biol.

[59] Epstein JI, Allsbrook WC, Amin MB, Egevad LL, Committee IG (2005). The 2005 International Society of Urological Pathology (ISUP) Consensus Conference on Gleason Grading of Prostatic Carcinoma. Am J Surg Pathol.

[60] Sorensen KD, Abildgaard MO, Haldrup C, Ulhoi BP, Kristensen H, Strand S (2013). Prognostic significance of aberrantly silenced ANPEP expression in prostate cancer. Br J Cancer.

