# Supplementary figures and images for: High levels of 5-hydroxymethylcytosine (5hmC) is an adverse predictor of biochemical recurrence after prostatectomy in ERG-negative prostate cancer
Source: Clin Epigenetics. 2015 Oct 15;7:111. doi: 10.1186/s13148-015-0146-5 (PMC4608326; doi:10.1186/s13148-015-0146-5)

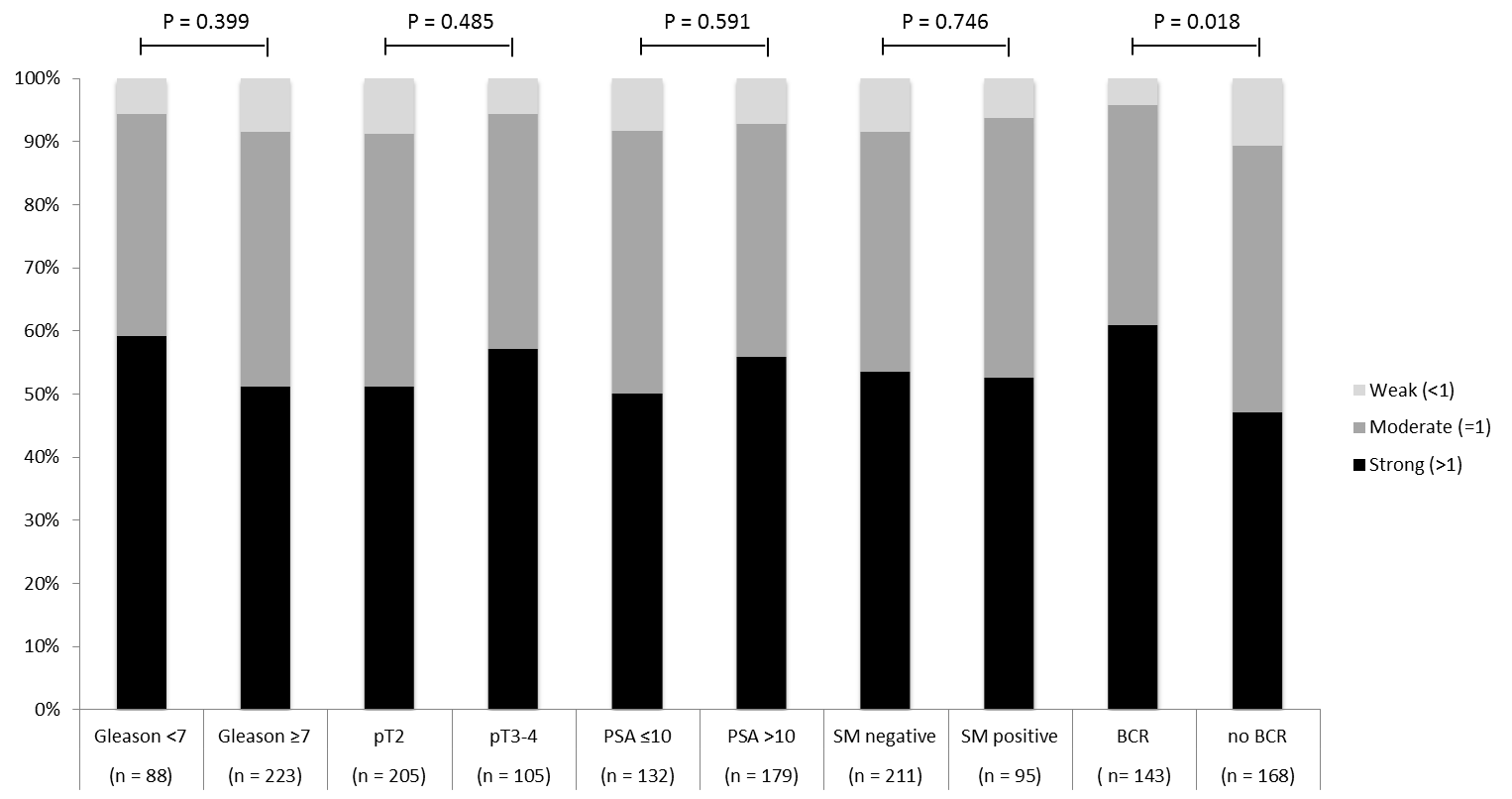

Supplement: Additional file 1: Figure S1. — Distribution of 5hmC scores by clinicopathological parameters and BCR in the full PC patient set. Full PC patient set, n = 311. P values: chi2 test. (TIFF 145 kb) [file 13148_2015_146_MOESM1_ESM.tif]

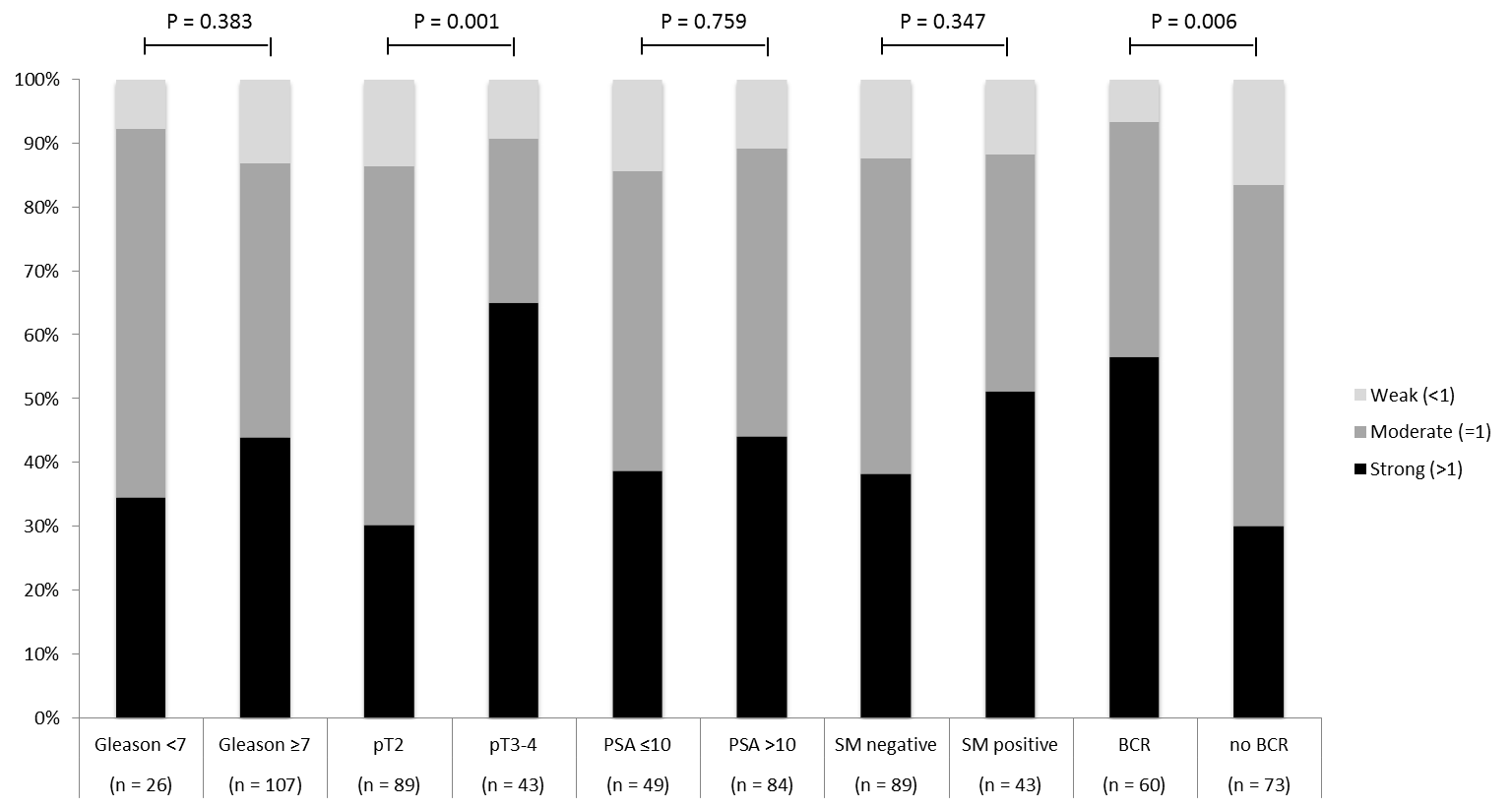

Supplement: Additional file 2: Figure S2. — Distribution of 5hmC scores by clinicopathological parameters and BCR status in ERG− PC. ERG− subset: n = 133. P values: chi2 test. (TIFF 146 kb) [file 13148_2015_146_MOESM2_ESM.tif]

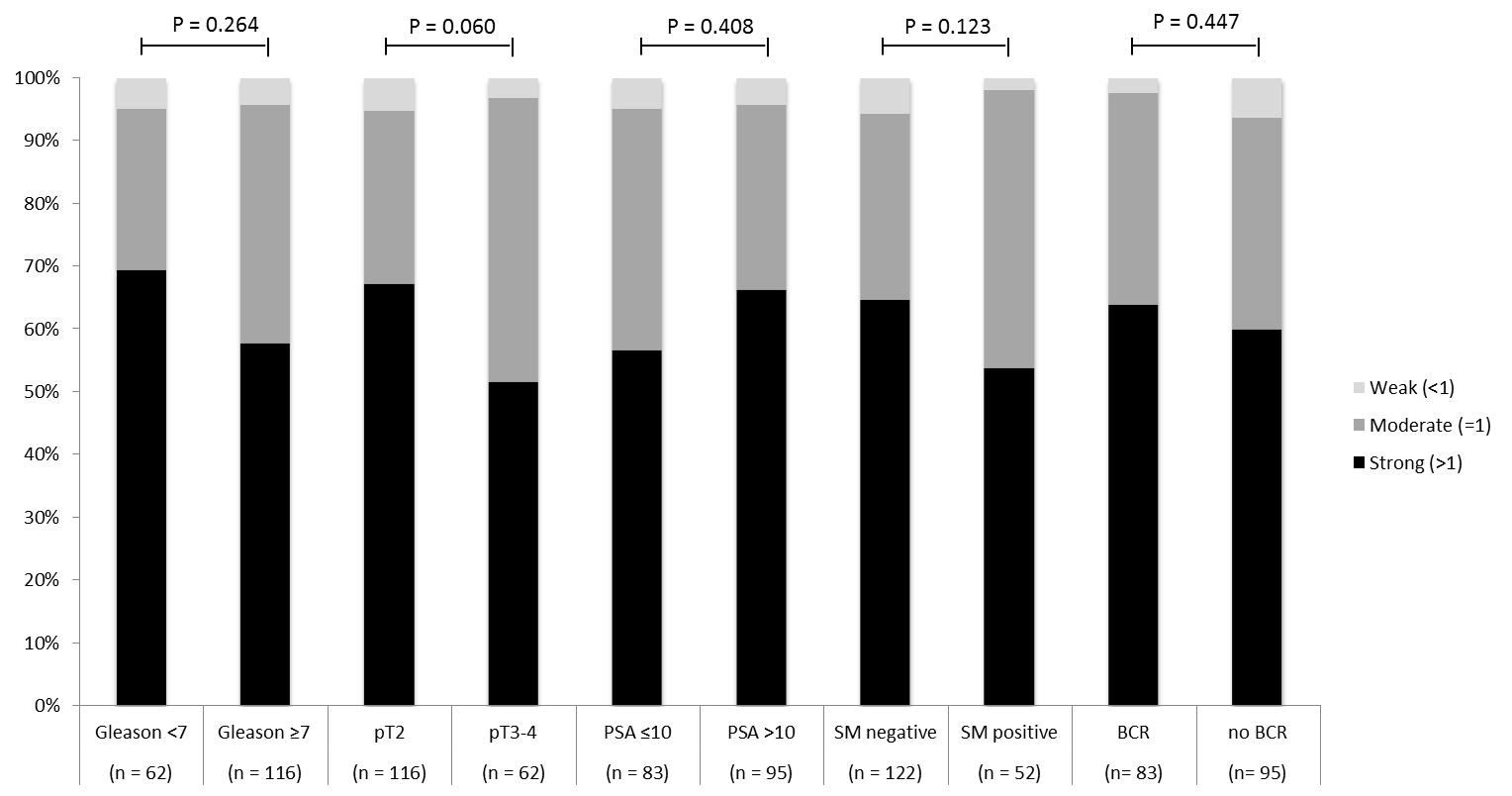

Supplement: Additional file 3: Figure S3. — Distribution of 5hmC scores by clinicopathological parameters and BCR status in ERG+ PC. ERG+ subset: n = 178. P values: chi2 test. (TIFF 143 kb) [file 13148_2015_146_MOESM3_ESM.tif]

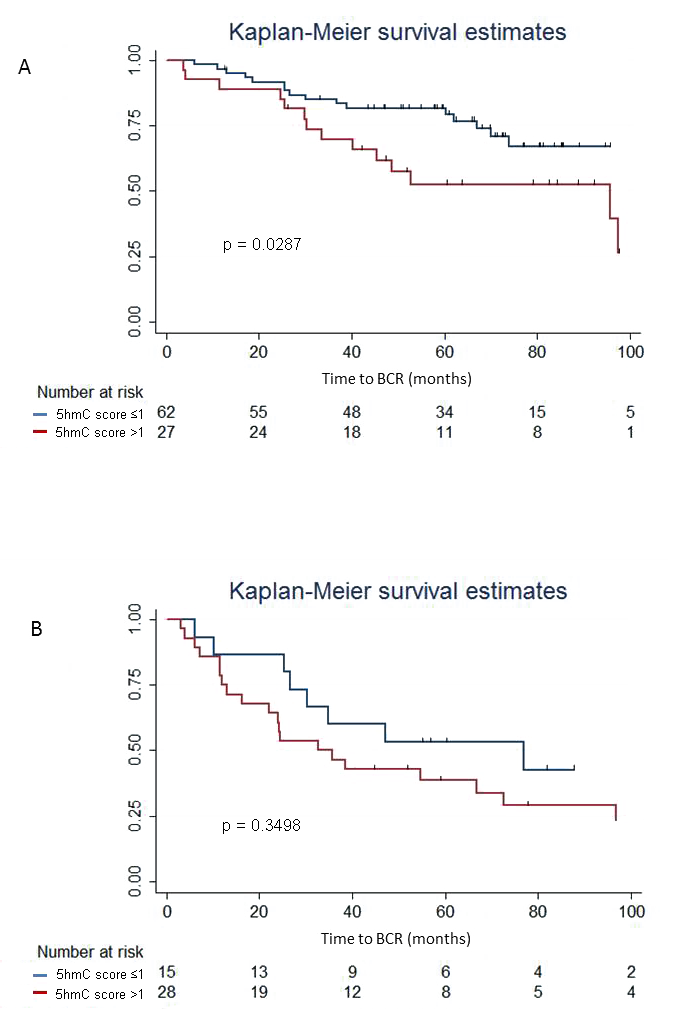

Supplement: Additional file 6: Figure S4. — Kaplan-Meier analysis: 5hmC score and time to BCR in ERG− PC stratified by pT stage. A: High 5hmC score (>1) was a significant adverse predictor of time to BCR in pT2 stage ERG− PCs (p = 0.029, log-rank test). B: The same trend was seen in pT3-4 stage ERG− PCs, but this was not statistically significant (p = 0.35, log-rank test). (TIFF 183 kb) [file 13148_2015_146_MOESM6_ESM.tif]
