# Supplementary material for: High levels of 5-hydroxymethylcytosine (5hmC) is an adverse predictor of biochemical recurrence after prostatectomy in ERG-negative prostate cancer
Source: Clin Epigenetics. 2015 Oct 15;7:111. doi: 10.1186/s13148-015-0146-5 (PMC4608326; doi:10.1186/s13148-015-0146-5)
Supplement: Additional file 4: Table S1. — Uni- and multivariate Cox regression analysis of BCR after RP in the full PC patient set. 5hmC analyzed as a dichotomized variable. (DOCX 33 kb) [file 13148_2015_146_MOESM4_ESM.docx]

Additional file 4: Table S1

| **All PCs (n = 311, 143 BCR)** | | | | | |
| --- | --- | --- | --- | --- | --- |
|  | **Univariate** | | | **Multivariate** | |
| **Variable** | **HR (95% CI)** | **p** | **C-index** | **HR (95% CI)** | **p** |
| 5hmC score (≤1 *vs.* >1) | 1.38 (0.99 - 1.94) | 0.059 | 0.55 | 1.38 (0.98 - 1.96) | 0.066 |
| Pre-op. PSA (≤ 10 *vs.* > 10) | 2.93 (2.00 - 4.29) | **<0.001** | 0.63 | 2.19 (1.47 - 3.26) | **<0.001** |
| Surgical margin (neg. *vs.* pos.) | 2.86 (2.04 - 4.00) | **<0.001** | 0.63 | 2.01 (1.40 - 2.90) | **<0.001** |
| Tumor stage (pT2 *vs.* pT3-4) | 2.96 (2.13 - 4.13) | **<0.001** | 0.64 | 1.88 (1.29 - 2.72) | **0.001** |
| Gleason score (≤ 6 *vs.* > 6) | 2.67 (1.70 - 4.17) | **<0.001** | 0.58 | 1.95 (1.22 - 3.12) | **0.005** |
