# Supplementary material for: High levels of 5-hydroxymethylcytosine (5hmC) is an adverse predictor of biochemical recurrence after prostatectomy in ERG-negative prostate cancer
Source: Clin Epigenetics. 2015 Oct 15;7:111. doi: 10.1186/s13148-015-0146-5 (PMC4608326; doi:10.1186/s13148-015-0146-5)
Supplement: Additional file 5: Table S2. — Uni- and multivariate Cox regression analysis of BCR after RP in ERG−/ERG+ PC patients. 5hmC analyzed as a dichotomized variable. a Global multivariate model including all parameters. b Final multivariate model including significant variables only. c Harrell’s C-index for final model including 5hmC. d Harrell’s C-index for final model excluding 5hmC. NA: not applicable. Significant p values (p < 0.05) are highlighted in bold. (DOCX 36 kb) [file 13148_2015_146_MOESM5_ESM.docx]

Additional file 5: Table S2

| ***ERG-* (n = 133, 60 BCR)** | | | | | | | | | |
| --- | --- | --- | --- | --- | --- | --- | --- | --- | --- |
|  | **Univariate** | | | **Multivariate^a^** | | **Multivariate^b^** | | | |
| **Variable** | **HR (95% CI)** | **p** | **C-index** | **HR (95% CI)** | **p** | **HR (95% CI)** | **p** | **C-index^c^** | **C-index^d^** |
| 5hmC score (≤1 *vs.* >1) | 2.11 (1.27 - 3.53) | **0.004** | 0.61 | 1.96 (1.13- 3-39) | **0.016** | 2.10 (1.25- 3.56) | **0.005** | 0.72 |  |
| Pre-op. PSA (≤ 10 *vs.* > 10) | 2.66 (1.44 - 4.92) | **0.002** | 0.60 | 2.38 (1.26 - 4.49) | **0.007** | 2.61 (1.40 - 4.84) | **0.002** |  | 0.68 |
| Surgical margin (neg. *vs.* pos.) | 2.83 (1.69 - 4.72) | **<0.001** | 0.62 | 2.49 (1.40 - 4.40) | **0.002** | 2.77 (1.65 - 4.63) | **<0.001** |  |  |
| Tumor stage (pT2 *vs.* pT3-4) | 2.26 (1.35 - 3.78) | **0.002** | 0.62 | 1.33 (0.729 - 2.42) | 0.353 | - | - |  |  |
| Gleason score (<7 *vs.* ≥7) | 2.22 (1.01 - 4.88) | **0.048** | 0.56 | 1.66 (0.739 - 3.73) | **0.22** | - | - |  |  |

| ***ERG+* (n = 178, 83 BCR)** | | | | | | | | | |
| --- | --- | --- | --- | --- | --- | --- | --- | --- | --- |
|  | **Univariate** | | | **Multivariate^a^** | | **Multivariate^b^** | | | |
| **Variable** | **HR (95% CI)** | **p** | **C-index** | **HR (95% CI)** | **p** | **HR (95% CI)** | **p** | **C-index^c^** | **C-index^d^** |
| 5hmC score (≤1 *vs.* >1) | 0.99 (0.63 - 1.55) | 0.952 | 0.50 | 1.06 (0.67 - 1.70) | 0.798 | - | - | NA |  |
| Pre-op. PSA (≤ 10 *vs.* > 10) | 3.19 (1.96 - 5.20) | **<0.001** | 0.65 | 2.01 (1.17 - 3.44) | **0.011** | 2.03 (1.26 – 3.25) | **0.004** |  | 0.76 |
| Surgical margin (neg. *vs.* pos.) | 2.91 (1.86 - 4.54) | **<0.001** | 0.63 | 1.74 (1.07 - 2.85) | **0.026** | 1.94 (1.25 – 3.01) | **0.003** |  |  |
| Tumor stage (pT2 *vs.* pT3-4) | 3.65 (2.35 - 5.68) | **<0.001** | 0.65 | 2.23 (1.35 - 3.70) | **0.002** | 2.13 (1.36 – 3.35) | **0.001** |  |  |
| Gleason score (<7 *vs.* ≥7) | 3.04 (1.76 - 5.27) | **<0.001** | 0.61 | 2.19 (1.22 - 3.92) | **0.009** | 2.76 (1.63 – 4.68) | **<0.001** |  |  |
